# Bacteraemia in a Nigerian hospital: Implementing antimicrobial resistance surveillance

**DOI:** 10.4102/jphia.v16i1.655

**Published:** 2025-02-07

**Authors:** Adewale A. Amupitan, Adeyemi T. Adeyemo, Adefunke O. Amupitan, Temitope O. Obadare, Aaron O. Aboderin

**Affiliations:** 1 Department of Medical Microbiology and Parasitology, Obafemi Awolowo University Teaching Hospitals Complex, Ile-Ife, Nigeria; 2 Department of Medical Microbiology and Parasitology, Basic Medical Sciences, Obafemi Awolowo University, Ile-Ife, Nigeria; 3 Department of Microbiology, Faculty of Pure and Applied Sciences, Kwara State University, Malete, Nigeria

**Keywords:** bacteraemia, antimicrobial-resistance, surveillance, healthcare-facility, Nigeria, GLASS, multidrug resistance, implementation

## Abstract

**Background:**

Surveillance of drug-resistant infections is crucial for antimicrobial resistance (AMR) control. Implementing surveillance in low- and middle-income countries (LMICs) is challenging.

**Aim:**

To investigate bacteraemia and describe AMR surveillance.

**Setting:**

Tertiary healthcare facility.

**Methods:**

Case finding was by WHO Global AMR and Use Surveillance System (GLASS). Blood samples were processed between May 2017 and June 2018, using BACTEC blood culture system. Bacterial identification, antibiotic susceptibility testing and detection of AMR genes followed standard protocols.

**Results:**

Aerobic blood cultures were conducted in a third of clinical sepsis cases (*n* = 601/1851), of which 114 (19.0%) were true positives, with a 2.2% contamination rate. Pathogens recovered included six priority blood pathogens reportable to WHO GLASS. Sixteen (30.2%) of 53 Gram-negative isolates were extended-spectrum beta-lactamase producers, predominantly harbouring *bla*_CTX-M,_ three (5.7%) were AmpC beta-lactamase producers, and 20 (37.7%) were carbapenem-resistant, predominantly harbouring *bla*_KPC_. Twenty-nine (50.9%) of 57 *Staphylococcus aureus* isolates were methicillin-resistant; 17 (58.6%) of these harboured *mec*A genes. Hospital-acquired infection (odds ratio [OR] = 0.3, 95% confidence interval [CI]=0.1–0.7, *p* = 0.004) was identified as a predisposing factor for the development of multidrug-resistant (MDR) bacteraemia. Bacteraemia with MDR organisms was significantly associated with mortality (OR = 3.8, 95% CI = 1.6–9.1, *p* = 0.001).

**Conclusion:**

A wide variety of bacteria are responsible for bacteraemia in our setting, with more than half being multidrug-resistant. Bacteraemia with multidrug-resistant organisms was significantly associated with mortality, hence, the need for this AMR surveillance initiative.

**Contribution:**

Implementing healthcare facility-based surveillance of AMR in LMICs is achievable despite limited microbiological laboratory capacity.

## Introduction

Healthcare facility-based surveillance of antimicrobial resistance (AMR) involves the collection, validation, interpretation and reporting of susceptibility data.^1^ Microbiological testing is a crucial tool for diagnosing bacterial infections and generating AMR data in low- and middle-income countries (LMICs). Optimising existing tools for diagnosing bacterial infections and generating bacterial identification and antimicrobial susceptibility data are the foundation of an AMR surveillance system.^2^ Bloodstream infection (BSI) is one of the most important causes of morbidity and mortality globally, with a higher burden in LMICs.^3^ Over time, AMR has made BSI a significant threat. A systematic analysis of the global burden of bacterial AMR published by the *Lancet* in 2022 estimated that in 2019, 1.27 million deaths were directly attributable to AMR, with 4.95 million deaths estimated to be associated with AMR.^4^ Western sub-Saharan Africa had the highest burden of all regions, with 27.3 deaths per 100 000 attributable to AMR.^4^ Although the increasing rate of AMR is a worldwide concern, these rates vary by region. Thus, regular surveillance is essential for monitoring the spectrum of bacterial pathogens implicated in infectious diseases like BSI and the trend of AMR among these pathogens in specific geographical locations. Such data are necessary to monitor emerging resistant strains of important pathogens and to facilitate the initiation of effective empirical therapy. Rational and correct use of antibiotics requires understanding common pathogens and drug resistance patterns in a particular region. However, improved capacity for medical microbiology diagnostics and surveillance is needed in LMICs to ensure effective surveillance, where in most cases, there is no antibiogram to inform local and institutional guidelines for rational prescription management of BSIs, and no baseline information to propel and sustain diagnostic and hospital stewardship.

In May 2015, the 68th World Health Assembly adopted the Global Action Plan (GAP) on AMR, which reflects the global consensus that AMR poses a profound threat to human health.^5^ One of the five strategic objectives of the GAP is to strengthen the evidence base through enhanced worldwide surveillance and research. The Global Antimicrobial Resistance and Use Surveillance System (GLASS) was developed to help the GAP on AMR.^6^ It is meant to be coordinated within countries’ national action plans. The Global Antimicrobial Resistance and Use Surveillance System aims to enable standardised, comparable and validated data on AMR to be collected, analysed and shared within countries to inform decision-making, drive local, national and regional action and provide evidence for action and advocacy.^5^ In 2017, Nigeria enrolled in the WHO GLASS. A national situation analysis and a National Action Plan (NAP) on AMR were developed. Using globally set criteria,^7^ two laboratories were selected as national reference laboratories (NRLs), and 10 laboratories (sentinel AMR laboratories) were engaged to participate in the National Antimicrobial Resistance Surveillance System in Nigeria.^8^ The NRLs receive a systematic referral of AMR isolates from the sentinel sites,^7^ including the Medical Microbiology Laboratory of the Obafemi Awolowo University Teaching Hospitals Complex (OAUTHC), Ile-Ife, Nigeria. The hospital was selected after fulfilling the essential requirements for setting up an AMR surveillance sentinel site, including successful participation in external quality assessment (EQA).

Owing to its high specificity in representing true infection and the fact that BSI is a low-hanging fruit to prioritise for AMR surveillance, blood for culture is the primary choice of sample required by GLASS. Blood culture to isolate the offending pathogen and determine its antimicrobial sensitivity pattern remains the mainstay of definitive diagnosis and management of BSI. Survival of patients with sepsis depends on the early administration of adequate empirical antimicrobial therapy. However, studies have reported that inappropriate treatment of BSI promotes the emergence of drug-resistant strains, among which is Wasihun et al.^9^

This study was conducted to investigate bacteraemia and describe AMR surveillance in OAUTHC, a tertiary healthcare facility and a sentinel site contributing to national AMR surveillance.

## Research methods and design

### Study design

This was a hospital-based descriptive cross-sectional study. Case finding, based on routinely collected clinical diagnostic specimens, was employed as described in the WHO Global AMR Surveillance System (GLASS).^5,6^ Cases were identified based on the microbiology results of blood samples collected and analysed as part of routine care between May 2017 and June 2018.

### Study population

The target population was all individuals with a clinical diagnosis of sepsis for which blood culture requests were made over the study period. All individuals with culture-positive bacterial BSIs and with features of clinical sepsis according to the Centers for Disease Control and Prevention (CDC) surveillance definitions^10^ were included in the study. We excluded cases with culture-positive BSIs caused by non-bacteria or bacteria of uncertain clinical relevance such as coagulase-negative staphylococci (if found only in one bottle),^10^ diphtheroids and *Bacillus* species.

### Demographic and clinical information

Relevant demographic and clinical information were obtained from individuals’ case notes with the aid of a proforma. The obtained information included age, sex, date of admission, location of the individual at the onset of BSI (home, ward), the probable source of bacteraemia and current antimicrobial therapy. In addition, the following predisposing factors or clinical conditions were also documented: intravascular catheter, indwelling urinary catheter, nasogastric tube, endotracheal tube, neutropenia, chemotherapy or surgery during the previous 30 days, organ transplantation, mechanical ventilation and total parenteral nutrition.

### Quality assurance for antimicrobial resistance surveillance

In 2017, we enrolled in the External Quality Assurance (EQA) system organised by the National Food Institute, Technical University of Denmark (DTU Food) in collaboration with partners and regional sites in the WHO Global Foodborne Infection Network. Other measures instituted to ensure quality specimen processing include routine internal quality controls and the use of standard operating procedures. Data management was done using WHONET software.

### Specimen collection, bacterial isolation and identification

Venous blood specimens obtained from clinically diagnosed cases of BSI were collected into two aerobic BACTEC blood culture broth media bottles (BD Bactec^TM^ Plus aerobic and Peds Plus aerobic, Becton Dickinson, Belgium), each containing 10 mL of blood (1 mL – 3 mL for the paediatric age group). Each bottle was loaded into the BACTEC 9050 semi-automated blood culture instrument (BACTEC™ 9050, Becton Dickinson, Belgium) following the manufacturer’s recommendation within 1 h of sample receipt at the clinical laboratory. Bottles flagged by the device for detectable levels of CO_2_ production had samples removed for Gram staining and subculturing onto chocolate agar (Oxoid, Basingstoke, Hants, United Kingdom) in 5% CO_2_, 5% sheep blood agar (Oxoid, Basingstoke, Hants, United Kingdom) and MacConkey agar (Oxoid, Basingstoke, Hants, United Kingdom) in an aerobic atmosphere. Gram-positive isolates were identified using standard microbiological techniques. *Staphylococcus aureus* and coagulase-negative staphylococci were identified using Gram staining, catalase test, coagulase test and growth on DNase agar (Oxoid, Basingstoke, Hants, United Kingdom) (DNA hydrolysis test). *Streptococcus* spp were identified using Gram staining, catalase test, optochin disc test and the haemolytic pattern on blood agar. *Enterococcus* spp were identified using Gram staining, catalase test and growth on bile esculin agar (Oxoid, Basingstoke, Hants, United Kingdom). Gram-negative isolates were identified based on their colonial morphology and Gram staining reaction and using the Microbact GNB 24E kit (Oxoid, Basingstoke, Hants, United Kingdom), which is a standardised micro-substrate system for identifying Enterobacterales and typical miscellaneous Gram-negative bacilli.

### Antibiotic susceptibility testing

Antibiotic susceptibility testing was carried out using the modified Kirby-Bauer disc diffusion technique recommended by the Clinical and Laboratory Standards Institute (CLSI) 2018.^11^ Quality control strains *Escherichia coli* ATCC 25922, *Pseudomonas aeruginosa* ATCC 27853 and *Staphylococcus aureus* ATCC 25923 were included in the experiments for quality assurance. Gram-positive isolates were tested using the following antibiotic discs (Oxoid, Basingstoke, Hants, United Kingdom): penicillin (10 µg), cefuroxime (30 µg), ceftriaxone (30 µg), co-amoxiclav (20 µ/10 µ), ciprofloxacin (5 µg), gentamicin (10 µg), erythromycin (15 µg), piperacillin-tazobactam (100 µg/10 µg) and vancomycin (30 µg). The vancomycin E-test, a gradient diffusion susceptibility method, involving the use of vancomycin (256 µg/mL – 0.015 µg/mL) minimum inhibitory (MIC) strip (Oxoid, Basingstoke, Hants, United Kingdom), was used to assess the susceptibility of *Staphylococcus aureus* to vancomycin. Gram-negative isolates were tested using the following antibiotic discs (Oxoid, Basingstoke, Hants, United Kingdom): ceftazidime (30 µg), ceftriaxone (30 µg), cefepime (30 µg), co-amoxiclav (20 µg/10 µg), ciprofloxacin (5 µg), gentamicin (10 µg), meropenem (10 µg), ertapenem (10 µg), cotrimoxazole (1.25 µg/23.75 µg), cefoxitin (30 µg) and piperacillin-tazobactam (100 µg/10 µg). The results were interpreted according to the CLSI guidelines and analysed using the WHONET application.^12^

### Determination of resistance phenotypes

Methicillin-resistant *Staphylococcus* species were identified by standard disc diffusion method using cefoxitin (30 µg) discs as described in the CLSI 2018 guidelines.^11^ Isolates with zones of inhibition ≤ 21 mm were recorded as methicillin-resistant *Staphylococcus aureus. S. aureus* ATCC 43300 and *S. aureus* ATCC 25923 were used as positive and negative controls, respectively.

A phenotypic confirmatory test to detect extended spectrum beta-lactamase (ESBL) production among Enterobacterales and other Gram-negative bacilli was performed using the combination disc test method with single discs of cefotaxime and ceftazidime and their respective combination discs with clavulanic acid. A 5 mm or more increase in the observed zones of inhibition for the combination discs compared to their respective single discs was taken as confirmatory evidence of ESBL production.^11^ *Klebsiella pneumoniae* ATCC 700603 and *Escherichia coli* ATCC 25922 were used as the positive and negative controls, respectively.

AmpC beta-lactamase production was detected using the Amp C disk test as described by Sinha et al. on isolates, which show resistance to two or more 3rd-generation cephalosporins and a beta-lactamase inhibitor.^13^ Briefly, we prepared lawn cultures of *E. coli* ATCC 25922 on Mueller-Hinton agar plates (Oxoid, Basingstoke, Hants, United Kingdom). Sterile discs (6 mm) were moistened with sterile saline and inoculated with several colonies of test organisms. The inoculated discs were placed besides cefoxitin discs (almost touching) on the inoculated plates. The plates were then incubated aerobically overnight at 35 °C. A positive test was indicated as flattening or indentation of the cefoxitin zone of inhibition in the vicinity of the test disc. A negative test had an undistorted zone of inhibition.

### Detection of resistance genes

Methicillin-resistant *Staphylococcus aureus* strains were assessed for the presence of the *mec*A gene. The Gram-negative bacilli were tested to detect the major ESBL genes, including *bla*_CTX-M_, *bla*_SHV_ and *bla*_TEM,_ and the carbapenemase genes, including *bla*_KPC_, *bla*_NDM_, *bla*_OXA-48_ and *bla*_VIM_, using polymerase chain reaction (Table 1).

**TABLE 1:** Primers and amplification reactions for targeted resistance genes.

Target gene	Primer sequence (5′ – 3′)	Amplicon size (bp)	Amplification reaction conditions	References
*mec*A	F: AAAATCGATGGTAAAGGTTGGC R: AGTTCTGGAGTACCGGATTTGC	533	Initial denaturation at 94 °C for 3 min 30 cycles of denaturation at 94 °C for 30 s, annealing at 55 °C for 30 s and extension at 72 °C for 30 s Final extension at 72 °C for 3 min	^14^
*bla*_TEM_	F: TTTCGTGTCGCCCTTATTCC R: ATCGTTGTCAGAAGTAAGTTGG	404	Initial denaturation at 94 °C for 45 s	^15^
*bla*_SHV_	F: CGCCTGTGTATTATCTCCCT R: CGAGTAGTCCACCAGATCCT	291	35 cycles of denaturation at 94 °C for 45 s, annealing at 60 °C for 30 s and extension at 72 °C for 1 min	
*bla*_CTX-M_	F: ATGGCGATTACTGGATAGATGG R: AGTCTTGGTCTTGGTTGTGAG	754	Final extension at 72 °C for 3 min	
*bla*_KPC_	F: CATTCAAGGGCTTTCTTGCTGC R: ACGACGGCATAGTCATTTGC	538	Initial denaturation at 95 °C for 15 mins	^16^
*bla*_NDM_	F: CCCGGCCACACCAGTGACA R: GTAGTGCTCAGTGTCGGCAT	129	35 cycles of denaturation at 94 °C for 30 s, annealing at 60 °C for 30 s and extension at 72 °C for 10 min	
*bla*_VIM_	F: GTTTGGTCGCATATCGCAAC R: AATGCGCAGCACCAGGATAG	382	Final extension at 72 °C for 10 min	
*bla*_OXA-48_	F: GCGTGGTTAAGGATGAACAC R: CATCAAGTTCAACCCAACCG	438		

Note: Please see the full reference list of the article, Amupitan AA, Adeyemo AT, Amupitan AO, Obadare TO, Aboderin AO. Bacteraemia in a Nigerian hospital: Implementing antimicrobial resistance surveillance. J Public Health Africa. 2025;16(1), a655. https://doi.org/10.4102/jphia.v16i1.655, for more information.

bp, base pairs.

### Data analysis and statistical techniques

Data analysis was performed using Statistical Package for Social Sciences (SPSS) version 20 (SPSS Inc., Chicago, Illinois, United States). Descriptive statistical analyses were carried out to summarise the demographic data, and the results are presented as frequency distributions, percentages, means and standard deviations, where appropriate. The risk factors associated with BSIs caused by multidrug-resistant bacteria were assessed using bivariate analysis with the chi-square test. *P*-values < 0.05 were considered statistically significant.

### Ethical considerations

Ethical approval was sought and obtained from the Ethics and Research Committee of OAUTHC, Ile-Ife, Nigeria (Project Research Number: NHREC/27/02/2009a. Protocol Number: ERC/2017/08/17). Written informed consent from individuals (and relatives of incapacitated adult individuals) was obtained for this study. In addition to obtaining written informed consent from parents or guardians of the young participants, assent was also obtained from youth old enough to give it. Individuals were de-identified, and their information was encoded with research-specific identification numbers. In addition, all information was stored in encrypted files and password-secured computer systems.

## Results

### Demographic distribution and clinical information of patients

There were 6519 admissions during the study period, of which 601 aerobic blood culture sets were obtained from 1851 cases of clinical sepsis. Of these 601 blood cultures, 114 (19.0%) were true positives, with a contamination rate of 2.2%. These 114 samples were obtained from 63 (55.3%) males and 51 (44.7%) females. Forty-five (39.5%) of the 114 individuals were neonates (< 1 month), 27 (23.7%) paediatric (1 month to < 18 years) and 42 (36.8%) were adults (≥ 18 years). Sixty-seven (58.8%) of the individuals had primary bacteraemia, and among the foci of BSIs identified, the urogenital system constituted the highest (*n* = 11, 9.6%). Other identified foci in the order of decreasing frequency included the central nervous system (*n* = 9, 7.9%), gastrointestinal system (*n* = 9, 7.9%), skin and soft tissues (*n* = 6, 5.3%), chest (*n* = 6, 5.3%), eyes (*n* = 4, 3.5%), cardiovascular system (*n* = 1, 0.9%) and bone (*n* = 1, 0.9%) (Table 2).

**TABLE 2:** Baseline clinical characteristics of individuals (*N* = 114).

Subject characteristics	Variable	*n*	%
Gender	Male	63	55.3
	Female	51	44.7
Age group	Neonates	45	39.5
	Paediatric	27	23.7
	Adults	42	36.8
Focus of infection	No focus identified	67	58.8
	Urogenital system	11	9.6
	Central nervous system	9	7.9
	Gastrointestinal system	9	7.9
	Surface ulcers/soft tissues	6	5.3
	Chest	6	5.3
	Eyes	4	3.5
	Cardiovascular system	1	0.9
	Bone	1	0.9

### Risk factors for the development of multidrug-resistant bacteraemia

One hundred and nine (95.6%) individuals were already on antibiotics before blood specimen collection, and 98.2% (*n* = 112) were on peripheral (IV) catheters. Diabetes mellitus (*n* = 9, 7.9%) was the most common co-morbidity among the individuals. Other co-morbidities include malignancy, haemoglobinopathy, chronic renal failure, chronic lung disease, burns/trauma, congenital anomalies and human immunodeficiency virus (HIV)/acquired immunodeficiency syndrome (AIDS) (Table 3). Neonatal variables were not statistically significant for the development of multidrug-resistant (MDR) bacteraemia. Bivariate analysis revealed that hospital-acquired infections (HAIs) (odds ratio [OR] = 0.4, 95% confidence interval [CI] = 0.2–0.7, *p* ≤ 0.001) and urethral catheter use (OR = 2.8, 95% CI = 1.0–7.7, *p* = 0.042) were factors associated with the development of MDR bacteraemia. However, further analysis with logistic regression identified only HAI (OR = 0.3, 95% CI = 0.1–0.7, *p* = 0.004) as the predisposing factor for the development of MDR bacteraemia.

**TABLE 3:** Association between clinical variables that influenced the development of multidrug-resistant bacteraemia.

Variables	*n*	%	Non-MDR		MDR		Bivariate analysis			Multivariate analysis			
			*n*	%	*n*	%	OR	95% CI	*p*	OR	95% CI	s.e.	*p*
**Gender (*N* = 114)**	-	-	-	-	-	-	0.7	0.4–1.14	0.170	1.4	0.6–3.5	0.5	0.432
Male	63	55.3	23	36.5	40	63.5	-	-	-	-	-	-	-
Female	51	44.7	25	49.0	26	51.0	-	-	-	-	-	-	-
**Age group (*N* = 114)**	-	-	-	-	-	-	0.7	0.4–1.1	0.126	0.5	0.2–1.6	0.6	0.248
Neonates	45	39.5	15	33.3	30	66.7	-	-	-	-	-	-	-
Paediatric	27	23.7	15	55.6	12	44.4	-	-	-	-	-	-	-
Adults	42	36.8	18	42.9	24	57.1	-	-	-	-	-	-	-
**Location and time of infection onset (*N* = 114)**	-	-	-	-	-	-	0.4	0.2–0.7	< 0.001	0.2	0.1–0.6	0.6	0.003
Hospital-acquired	53	46.5	12	22.6	12	77.4	-	-	-	-	-	-	-
Community-acquired	61	53.5	36	59.0	36	41.0	-	-	-	-	-	-	-
**Antibiotic use (*N* = 114)**	-	-	-	-	-	-	-	-	-	-	-	-	-
Before blood specimen collection	109	95.6	46	42.2	46	57.8	0.9	0.1–5.7	1.000	NT	-	NT	NT
**Invasive procedures/surgery (*N* = 114)**	-	-	-	-	-	-	-	-	-	-	-	-	-
Intravascular catheters	112	98.2	48	42.9	48	57.1	-	-	0.508	NT	-	NT	NT
Urethral catheter use	25	21.9	6	24.0	6	76.0	2.8	1.0–7.7	0.042	0.3	0.0–1.4	0.9	0.113
Nasogastric Tube	4	3.5	0	0.0	0	100.0	-	-	0.137	NT	-	NT	NT
Endotracheal tube use	1	0.9	0	0.0	0	100.0	-	-	1.000	NT	-	NT	NT
Central venous catheter	1	0.9	0	0.0	0	100.0	-	-	1.000	NT	-	NT	NT
Prosthesis	1	0.9	0	0.0	0	100.0	-	-	1.000	NT	-	NT	NT
Surgery	7	6.1	1	14.3	1	85.7	4.7	0.5–40.4	0.236	0.7	0.0–11.8	1.5	0.794
Dialysis	2	1.8	1	50.0	1	50.0	0.7	0.0–11.9	1.000	NT	-	NT	NT
**Comorbidities/immunocompromised conditions (*N* = 114)**	-	-	-	-	-	-	-	-	-	-	-	-	-
Diabetes mellitus	9	7.9	5	55.6	5	44.4	0.6	0.1–2.2	0.489	22.6	1.1–444.8	1.5	0.041
Malignancy	7	6.1	2	28.6	2	71.4	1.9	0.4–10.2	0.697	1.4	0.2–13.0	1.1	0.767
Haemoglobinopathy	6	5.3	3	50.0	3	50.0	0.7	0.1–3.7	0.695	NT	-	NT	NT
Chronic renal failure	4	3.5	1	25.0	1	75.0	2.2	0.2–22.2	0.637	0.1	0.0–3.2	1.5	0.178
Chronic lung disease	2	1.8	0	0.0	0	100.0	-	-	0.508	NT	-	NT	NT
Burns/Trauma	2	1.8	1	50.0	1	50.0	0.7	0.0–11.9	1.000	NT	-	NT	NT
Congenital anomalies	2	1.8	1	50.0	1	50.0	0.7	0.0–11.9	1.000	NT	-	NT	NT
HIV/AIDS	1	0.9	0	0.0	0	100.0	-	-	1.000	NT	-	NT	NT
**Neonatal risk factors (*N* = 45)**	-	-	-	-	-	-	-	-	-	-	-	-	-
Prematurity	13	28.9	4	30.8	4	69.2	1.2	0.3–4.7	1.000	NT	-	NT	NT
Low birth weight	13	28.9	3	23.1	3	76.9	2.0	0.5–8.7	0.492	NT	-	NT	NT
Birth asphyxia	10	22.2	1	10.0	1	90.0	6.0	6.8–52.8	0.129	0.4	0.2–1.3	0.6	0.140

NT, not tested; OR, odds ratio; CI, confidence interval; s.e., standard error; MDR, multidrug-resistant; HIV, human immunodeficiency virus; AIDS, acquired immunodeficiency syndrome.

### Distribution of identified bloodstream bacterial pathogens

A total of 114 bacterial pathogens were recovered from the bloodstream, with one bacterium isolated in each of the 114 samples. There was no co-infection. Gram-positive bacteria constituted 53.5% (*n* = 61) of all isolates, while Gram-negative bacteria constituted 46.5% (*n* = 53) (Table 4). *Staphylococcus aureus* (*n* = 57, 50%) was the most common bacteria recovered, followed by *Klebsiella pneumoniae* (*n* = 18, 15.8%). Others included *Escherichia coli* (*n* = 6, 5.3%), *Salmonella* spp. (*n* = 6, 5.3%), *Stenotrophomonas maltophilia* (*n* = 6, 5.3%), *Enterobacter* spp. (*n* = 4, 3.5%), *Acinetobacter* spp. (*n* = 4, 3.5%), *Pseudomonas* spp. (*n* = 4, 3.5%), *Enterococcus* spp. (*n* = 3, 2.6%), *Morganella morganii* biogroup 1 (*n* = 2, 1.8%), *Streptococcus pneumoniae* (*n* = 1, 0.9%), *Hafnia alvei* (*n* = 1, 0.9%), *Photorhabdus asymbiotica* (*n* = 1, 0.9%) and *Serratia liquifaciens* complex (*n* = 1, 0.9%). Majority of the *Staphylococcus aureus* (*n* = 27, 47.4%) and *Klebsiella* species (*n* = 7, 38.9%) were isolated from the neonates. On the other hand, the *Salmonella* species were mostly isolated from patients in the paediatric age group (*n* = 5, 83.3%), while *Escherichia coli* (*n* = 4, 66.7%) were mostly isolated from adults (Table 4).

**TABLE 4:** Distribution of identified bloodstream bacterial pathogens.

Bacterial pathogens	All (*n*)	Neonates (*n* = 45)		Paediatric (*n* = 27)		Adults (*n* = 42)	
		*n*	%	*n*	%	*n*	%
**Gram-positive**	**61**	**28**	**45.9**	**13**	**21.3**	**20**	**32.8**
*Staphylococcus aureus*	57	27	47.4	11	19.3	19	33.3
*Enterococcus* spp.	3	1	33.3	1	33.3	1	33.3
*Streptococcus pneumoniae*	1	0	0.0	1	100.0	0	0.0
**Gram-negative**	**53**	**17**	**32.1**	**14**	**26.4**	**22**	**41.5**
*Klebsiella* spp.	18	7	38.9	5	27.8	6	33.3
*Escherichia coli*	6	2	33.3	0	0.0	4	66.7
*Salmonella* spp.	6	0	0.0	5	83.3	1	16.7
*Stenotrophomonas maltophilia*	6	2	33.3	2	33.3	2	33.3
*Enterobacter* spp.	4	2	50.0	0	0.0	2	50.0
*Acinetobacter* spp.	4	2	50.0	1	25.0	1	25.0
*Pseudomonas* spp.	4	1	25.0	0	0.0	3	75.0
*Morganella morganii* biogroup 1	2	1	50.0	0	0.0	1	50.0
*Hafnia alvei*	1	0	0.0	1	100.0	0	0.0
*Photorhabdus asymbiotica*	1	0	0.0	0	0.0	1	100.0
*Serratia liquifaciens* complex	1	0	0.0	0	0.0	1	100.0

### Antibiotic resistance patterns of the isolates

Half (*n* = 29, 50.9%) of the *Staphylococcus aureus* isolates were methicillin-resistant, two (66.7%) of the *Enterococcus* species isolates were vancomycin-resistant, while the only *Streptococcus pneumoniae* isolate showed good susceptibility to the tested antibiotics (Table 5).

**TABLE 5:** Antibiotic resistance pattern among Gram-positive bacteria.

Antibiotics/phenotype	Number (percentage) of resistant isolates							
	*Staphylococcus aureus* (*n* = 57)		*Streptococcus pneumoniae* (*n* = 1)		*Enterococcus* spp (*n* = 3)		All Gram-positive isolates (*n* = 61)	
	*n*	%	*n*	%	*n*	%	*n*	%
Penicillin G	57	100.0	0	0	3	100.0	60	98.4
Amoxicillin-Clavulanate	31	54.4	0	0	2	66.7	33	54.1
Ampicillin	NT	NT	NT	NT	3	100.0	3	4.9
Piperacillin-Tazobactam	20	35.1	0	0	1	33.3	21	34.4
Cefoxitin	29	50.9	0	0	3	100.0	31	50.8
Ceftriaxone	37	64.9	0	0	3	100.0	40	65.6
Cefuroxime	30	52.6	0	0	3	100.0	33	54.1
Ciprofloxacin	25	43.9	0	0	3	100.0	28	45.9
Erythromycin	18	31.6	0	0	3	100.0	21	34.4
Gentamicin	18	31.6	0	0	2	66.7	20	32.8
Vancomycin	0	0.0	0	0	2	66.7	2	3.3
MDR	22	38.6	0	0	3	100.0	25	41.0
MRSA	29	50.9	NT	NT	NT	NT	29	47.5
VISA / VRE	8	14.0	NT	NT	2	66.7	10	16.4

NT, not tested; MDR, multidrug resistance; MRSA, methicillin-resistant *Staphylococcus aureus*; VISA, vancomycin intermediate-resistant *Staphylococcus aureus*; VRE, vancomycin-resistant *Enterococcus*.

There was high resistance to the cephalosporins, aminoglycosides and fluoroquinolones, while low resistance was observed to the extended-spectrum penicillins and the carbapenems (Table 6).

**TABLE 6:** Antibiotic resistance pattern among Gram-negative bacteria.

Antibiotics/ phenotype	Number and percentage of resistant isolates																							
	*Escherichia coli* (*n* = 6)		*Klebsiella* spp (*n* = 18)		*Enterobacter* spp (*n* = 4)		*Salmonella* spp (*n* = 6)		*Morganella morganii* (*n* = 2)		*Hafnia alvei* (*n* = 1)		*Serratia liquefaciens* (*n* = 1)		*Photorhabdus asymbiotica* (*n* = 1)		*Pseudomonas* spp (*n* = 4)		*Acinetobacter* spp (*n* = 4)		*Stenotrophomonas maltophilia* (*n* = 6)		All GNB tested (*n* = 53)	
	*n*	%	*n*	%	*n*	%	*n*	%	*n*	%	*n*	%	*n*	%	*n*	%	*n*	%	*n*	%	*n*	%	*n*	%
Amoxicillin-Clavulanate	6	100.0	18	100.0	2	50.0	2	33.3	1	50.0	1	100.0	1	100.0	0	0.0	4	100.0	3	75.0	2	33.3	40	75.5
Ampicillin	6	100.0	NT	NT	NT	NT	NT	NT	NT	NT	NT	NT	NT	NT	NT	NT	NT	NT	NT	NT	NT	NT	6	11.3
Piperacillin-Tazobactam	0	0.0	6	33.3	1	25.0	0	0.0	0	0.0	1	100.0	1	100.0	0	0.0	2	50.0	1	25.0	1	16.7	13	24.5
Cefepime	5	83.3	16	88.9	2	50.0	1	16.7	1	50.0	1	100.0	1	100.0	1	100.0	4	100.0	3	75.0	6	100.0	41	77.4
Cefotaxime	6	100.0	18	100.0	3	75.0	2	33.3	1	50.0	1	100.0	1	100.0	1	100.0	4	100.0	4	100.0	5	83.3	46	86.8
Ceftazidime	3	50.0	18	100.0	3	75.0	0	0.0	0	0.0	1	100.0	1	100.0	1	100.0	3	75.0	3	75.0	6	100.0	39	73.6
Ceftriaxone	3	50.0	16	88.9	2	50.0	1	16.7	1	50.0	1	100.0	1	100.0	0	0.0	3	75.0	3	75.0	4	66.7	35	66
Cefoxitin	0	0.0	4	22.2	0	0.0	1	16.7	2	100.0	1	100.0	1	100.0	0	0.0	4	100.0	2	50.0	4	66.7	19	35.8
Ciprofloxacin	4	66.7	15	83.3	2	50.0	1	16.7	1	50.0	1	100.0	1	100.0	1	100.0	4	100.0	2	50.0	1	16.7	33	62.3
Gentamicin	2	33.3	16	88.9	2	40.0	1	16.7	1	50.0	1	100.0	1	100.0	0	0.0	2	50.0	2	50.0	1	16.7	29	54.7
Ertapenem	1	16.7	6	33.3	0	0.0	0	0.0	0	0.0	1	100.0	1	100.0	1	100.0	4	100.0	4	100.0	4	66.7	22	41.5
Meropenem	2	33.3	3	16.7	0	0.0	1	16.7	0	0.0	1	100.0	0	0.0	1	100.0	2	50.0	0	0.0	NT	16.0	30.2	-
Trimethoprim-Sulfamethoxazole	5	83.3	17	94.4	4	100.0	3	50.0	1	50.0	1	100.0	1	100.0	1	100.0	4	100.0	0	0.0	1	16.7	38	71.7
**MDR**	5	83.3	18	100.0	2	50.0	1	16.7	1	50.0	1	100.0	1	100.0	1	100.0	4	100.0	3	75.0	4	66.7	41	77.4
**ESBL**	2	33.3	10	55.6	3	75.0	0	0.0	1	50.0	0	0.0	0	0.0	0	0.0	0	0.0	0	0.0	0	0.0	16	30.2
**AmpC**	0	0.0	0	0.0	0	0.0	0	0.0	2	100.0	0	0.0	1	100.0	0	0.0	0	0.0	0	0.0	0	0.0	3	5.7
**Carbapenem resistance**	2	33.3	8	44.4	0	0.0	1	16.7	0	0.0	1	100.0	0	0.0	1	100.0	4	100.0	3	75.0	0	0.0	20	37.7

NT, not tested; GNB, gram-negative bacilli; MDR, multidrug resistance; ESBL, extended spectrum β-lactamase; AmpC, AmpC β-lactamase.

### Prevalence and patterns of multidrug resistance phenotypes

Sixty-six (58%) of the isolates were resistant to at least three different classes of antibiotics combined (multidrug resistant), including 41% (*n* = 25) of the Gram-positive bacteria and 77.4% (*n* = 41) of the Gram-negative bacteria. All *Enterococcus* species isolates (*n* = 3, 100%) were MDR, while 22 (38.6%) of the *Staphylococcus aureus* isolates were MDR (Table 5). Among the Gram-negative bacteria, all (100%) of the *Pseudomonas* species (*n* = 4), *Klebsiella* species (*n* = 18), *Hafnia alvei* (*n* = 1), *Photorhabdus asymbiotica* (*n* = 1) and *Serratia liquifaciens* (*n* = 1) isolates were MDR. Of the remaining Gram-negative bacteria, 83.3% of the *Escherichia coli* isolates (*n* = 5), 75.0% of *Acinetobacter* species (*n* = 3), 66.7% of *Stenotrophomonas maltophilia* (*n* = 4), 50.0% of *Enterobacter* species (*n* = 2), 50% of *Morganella* species (*n* = 1) and 16.7% of the *Salmonella* species (*n* = 1) isolates were MDR (Table 6).

Of the 57 *Staphylococcus aureus* isolates, 51% (*n* = 29) were methicillin-resistant, while 14% (*n* = 8) had intermediate susceptibility to vancomycin (Table 5). Two of the three (66.7%) *Enterococcus* species were vancomycin-resistant. Of the 53 Gram-negative bacilli, 16 (30.2%) were ESBL producers, three (5.7%) were ampC-β-lactamase producers and 20 (37.7%) were carbapenem-resistant (Table 6).

Of the 18 *Klebsiella pneumoniae* isolates, 10 (55.6%) were ESBL producers, while eight (44.4%) were carbapenem-resistant. Of the six *Escherichia coli*, two (33.3%) were ESBL producers and two (33.3%) were carbapenem-resistant. One (16.7%) of the *Salmonella* species isolates, three (75%) of the *Acinetobacter* species isolates and all four *Pseudomonas* species isolates were carbapenem-resistant. Of the four *Enterobacter* species, three (75%) were ESBL producers, while one (50%) of the two *Morganella morganii* isolates was an ESBL producer, and the two were ampC-β-lactamase producers. The only *Hafnia alvei* and *Photorhabdus asymbiotica* isolates were carbapenem-resistant, and the only *Serratia liquefaciens* complex isolate was an ampC-β-lactamase producer (Table 6).

### Resistance genes detected among the isolates

Seventeen of the 29 (58.6%) *Staphylococcus aureus* isolates that were phenotypically methicillin-resistant were positive for the *mecA* gene. Sixteen of the 53 (30.2%) Gram-negative bacteria were phenotypically confirmed to be ESBL producing, having at least one ESBL gene. *bla*_CTX-M_ (*n* = 13, 81.3%) was the most common gene detected among these ESBL-producing isolates, followed by *bla*_TEM_ (*n* = 12, 75%) and *bla*_SHV_. (*n* = 12, 75%). Twenty of the 53 (37.7%) Gram-negative bacteria phenotypically resistant to ertapenem or meropenem had at least one carbapenemase gene, with *bla*_KPC_ (*n* = 14, 70%) being the most common gene detected, followed by *bla*_VIM_ (*n* = 7, 35%), *bla*_NDM_ (*n* = 5, 25%) and *bla*_OXA-48_ (*n* = 2, 10%). *Klebsiella pneumoniae* accounted for 40% (*n* = 8) of these isolates, followed by *Pseudomonas* spp. (*n* = 4, 20%) and *Acinetobacter* spp. (*n* = 3, 15%).

### Mortality from multidrug-resistant bacteraemia

Overall, MDR bacteraemia is significantly associated with mortality, with four times greater odds of death (OR = 3.8, 95% CI = 1.6–9.1, *p* = 0.001) in patients with MDR bacteraemia compared to those who did not have MDR bacteraemia.

## Discussion

This surveillance focuses on isolates from blood, a priority specimen for GLASS.^5,8^ Over the study period, a wide variety of bacteria were responsible for bacteraemia, with more than half being multidrug-resistant. The spectrum of pathogens responsible for BSI is highly variable geographically and over time.^17^ In this study, there were almost equal proportions of Gram-positive (53.5%) and Gram-negative (46.5%) BSI pathogens. Among the pathogens recovered were six of the priority pathogens from blood reportable to WHO GLASS,^5,8^ namely *Staphylococcus aureus, Klebsiella pneumoniae, Escherichia coli, Salmonella* spp., *Acinetobacter* spp. and *Streptococcus pneumoniae. Staphylococcus aureus* and *Klebsiella* spp. were more commonly isolated from neonates, while *Salmonella* spp. was most common among paediatric age group. Among adults, *Escherichia coli* was the most common pathogen isolated. A preponderance of *Staphylococcus aureus* and *Klebsiella pneumoniae* in the neonatal age group was likewise reported by Mokuolu et al.^18^ in Nigeria and another study from sub-Saharan Africa.^19^ These findings have implications for the choice of empiric antibiotic treatment in these age groups.

Among the Enterobacterales in this study, *Klebsiella* spp, *Escherichia coli* and *Enterobacter* spp showed high resistance to the commonly prescribed antibiotics, including the third-generation cephalosporins. However, *Salmonella* isolates in this study showed low resistance to the major classes of antibiotics, similar to the findings of a systematic review on AMR in West Africa.^20^ On the contrary, some studies done in Asia have reported high resistance rates among *Salmonella*.^21,22^ These *Salmonella* isolates showed high rates of MDR^22^ with high rates of decreased ciprofloxacin susceptibility.^21,22^ Among the non-fermentative isolates, high resistance was noted for *Pseudomonas* spp and *Acinetobacter* spp to ceftazidime, cefepime, ciprofloxacin and gentamicin. These results agree with the findings by Phe et al.^22^ and Grewal et al.^23^ It is also noteworthy that *Stenotrophomonas maltophilia* showed high resistance to cefepime (100%) and third-generation cephalosporins (67% – 100%). This finding is not unexpected because antibiotic resistance is reportedly pronounced in *Stenotrophomonas*.^23^ The implication of these findings is that for effective treatment of BSI, there might be dependence on last-resort antibiotics, with attendant antibiotic stewardship issues.

Among the Gram-positive bacteria, *Staphylococcus aureus* showed high resistance to first-line agents, including ceftriaxone, amoxicillin-clavulanate, cefuroxime and ciprofloxacin. Reports from other studies show comparable resistance rates, among which is the SENTRY Antimicrobial Surveillance.^17^ Half of the *Staphylococcus aureus* recovered from blood in this study were methicillin-resistant. Elevated methicillin resistance rates among *Staphylococcus aureus* recovered from blood have been reported globally, and this is associated with significant morbidity and mortality.^24^ Between 30% and 40% of *S. aureus* isolates from blood in the United States are methicilin-resistant *Staphylococcus aureus* (MRSA).^17^ This proportion ranges between 12.8% and 26.3% in Europe and is greater than 60% in the Asia-Pacific region (Taiwan, Singapore, Japan and Hong Kong).^17^ This finding has implication for the choice of antibiotic treatment for BSI caused by Gram-positive bacteria. Although no vancomycin resistance was observed in this study, possibly because of its low prescription rate in this setting, eight (14.0%) of the MRSA isolates had intermediate susceptibility to vancomycin (MIC = 4 µg/mL). This finding is noteworthy because these strains may become vancomycin-resistant, especially in life-threatening invasive infections where vancomycin is commonly indicated.

Fifty-eight per cent (*n* = 66/114) of the recovered bacteria were multi-drug resistant, of which 41% (*n* = 25/61) were Gram positive and 77.4% (*n* = 41/53) were Gram negative. This pattern is not surprising as high resistance rates have been reported among bacterial pathogens in all WHO regions, with sub-Saharan Africa having the highest estimated AMR burden.^4^ In this study, ESBL phenotypes were observed in 30% of Gram-negative bacilli. These results are comparable to findings by Tian et al.^25^ The high proportion of *bla*_CTX-M_ found in this study is consistent with previous findings that *bla*_CTX-M_ is the most prevalent ESBL gene among Enterobacterales worldwide.^26^ The global dissemination of this gene is primarily attributed to mobile genetic elements that harbour the genes and frequent co-existence of these genes with genes conferring resistance to the other classes of antibiotics like fluoroquinolones and aminoglycosides, leading to a high rate of co-selection. It is also noteworthy that prior exposure to cephalosporins is an independent risk factor for individuals developing bacteraemia caused by ESBL-producing Gram-negative bacilli (GNB).^27^ About 37% (*n* = 20) of GNBs in this study were phenotypically resistant to ertapenem or meropenem, with a substantial number of them (*n* = 14) harbouring at least one type of carbapenemase gene, including *bla*_KPC_ (70.0%), *bla*_VIM_ (35.0%), *bla*_NDM_ (25.0%) and *bla*_OXA-48_ (10.0%). Similar findings were reported by Pang et al.^28^

One of the critical findings in this study was that 95.6% (*n* = 109) of individuals recruited were already on antibiotics before blood specimen collection. This action can cause selective pressure, allowing antibiotic-resistant bacteria to survive and multiply, as seen in the pattern of bacteria involved in bacteraemia, which are substantially multidrug-resistant. In this study, urethral catheter use was a statistically significant factor associated with the development of MDR bacteraemia. This finding is not surprising as the focus of infection that contributed mainly to the development of BSI in this study was the urogenital system. This finding agrees with the study done by Swati et al.,^29^ who found the presence of urinary catheters as an independent risk factor and predictor for the development of MDR GNB bacteraemia. Furthermore, as found in this study, HAI was found to be a predisposing factor for the development of MDR bacteraemia. This could possibly explain the four times greater odds of death in individuals with MDR bacteraemia compared to those who did not have MDR bacteraemia, as HAIs are often associated with poor treatment outcomes.

This baseline study on bacteraemia provided the impetus for AMR surveillance in this facility. Before now, the surveillance set-up was paper-based, and there were challenges of limited microbiological laboratory capacity, poor data management system, limited funding and a lack of personnel. To mitigate some of these challenges and ensure effective AMR surveillance, the facility keyed into the existing WHO GLASS network through the Nigerian National Coordinating Centre (Nigeria Centre for Disease Control [NCDC]) in 2017.^8^ Since then, there has been responsibility for the institutional supply of surveillance data to the global network. Obafemi Awolowo University Teaching Hospitals Complex, being one of the sentinel sites in Nigeria’s AMR surveillance activities,^7^ received a boost through the Fleming Fund support (2019–2021).^30^ The Fleming Fund revamped the microbiology laboratory diagnostic system in the facility and 10 other facilities in the country: engaged in the physical renovation of physical structures, supply of laboratory equipment and consumables and workforce capacity building. Before this conscious effort to implement a surveillance system, the laboratory management system was poor. However, for the effectiveness of ongoing surveillance, the institution enrolled in EQA systems initially provided by the Technical University of Denmark (DTU Food) through a global network in 2017 and currently by EQuAFRICA as coordinated by the NCDC. Some of the current initiatives in the facility for the sustenance of effective surveillance include ensuring the buy-in of clinicians to improve requests for blood culture, that is, clinician-initiated/directed blood culture requests, especially before initiation of empirical antibiotic therapy. Other initiatives include strengthening antimicrobial stewardship in the facility and ensuring the development of evidenced-based local guidelines (using the generated antibiogram) for clinicians to follow in prescribing antimicrobials for the management of BSIs. As part of the drive to improve AMR surveillance, the institution enrolled as one of the 15 hospitals for the implementation of ACORN II (A Clinically Oriented Antimicrobial Resistance Network) across nine Asian and West African countries.^31^ This experience stood the healthcare facility in good stead to becoming one of the pilot sites for Wellcome Trust-funded SEDRILIMS (The Surveillance and Epidemiology of Drug-Resistant Infections Laboratory Information Management System) in Africa.

It is important to acknowledge that the bacteriological profile of individuals with clinical sepsis might have changed with the data generated 6 years ago; this study brings to the fore, the high burden of AMR as an emerging threat to global health.

### Limitations

Anaerobic bacteria implicated in BSI could not be recovered because of a lack of diagnostic capacity. Also, there was low sensitivity of blood cultures in recovering bloodstream bacterial pathogens, especially for individuals who had prior antibiotic treatment.

## Conclusion

This study shows that a wide variety of bacteria are responsible for bacteraemia in our setting, with more than half being multidrug-resistant. It also revealed that bacteraemia with multidrug-resistant organisms was significantly associated with mortality. This underscores the need for this AMR surveillance initiative. Though the implementation was found to be challenging, this study demonstrated that implementation of healthcare facility-based surveillance of AMR in LMICs is achievable despite limited microbiological laboratory capacity. We expect our findings to drive the need for infection prevention and control and antimicrobial stewardship interventions to manage BSIs. Also, we hope that following this study, surveillance of AMR will be sustained and expanded for continuous monitoring of resistance trends.
